# Atypical Teratoid/Rhabdoid Tumor of the Central Nervous System in Children: Case Reports and Literature Review

**DOI:** 10.3389/fsurg.2022.864518

**Published:** 2022-05-16

**Authors:** Gengyin Guo, Jianfeng Zhuang, Keke Zhang, Zhizhen Zhou, Yanjun Wang, Zhen Zhang

**Affiliations:** ^1^ Department of Neurosurgery, Shandong Provincial Hospital Affiliated to Shandong First Medical University, Jinan, Shandong, China; ^2^ Department of Neurosurgery, Qilu Hospital of Shandong University, Jinan, China; ^3^ Department of Otolaryngology, Shandong Provincial Hospital Affiliated to Shandong First Medical University, Jinan, Shandong, China; ^4^ Department of Neurosurgery, Weishan People’s Hospital, Jining, Shandong, China; ^5^ State Key Laboratory of Translational Medicine and Innovative Drug Development, Jiangsu Simcere Pharmaceutical Co., Ltd., Nanjing, China; ^6^ Shandong University of Traditional Chinese Medicine, First Clinical College, Jinan, China

**Keywords:** atypical teratoid/rhabdoid tumor, pediatric brain tumors, integrase interactor 1, adjuvant therapy, molecular subgrouping

## Abstract

Atypical teratoid/rhabdoid tumor (AT/RT) of the central nervous system is a highly malignant tumor that mainly occurs in children under the age of 3 and has only been rarely described in adults. The fact that AT/RT patients have such a terrible prognosis is even more regrettable. Herein, we reported two special cases of AT/RT, both of which were under 3 years. Symptoms at presentation included increased intracranial pressure and cerebellar symptoms such as headache, altered gait, and ataxia. As for the tumor location, one was infratentorial in the posterior fossa, and the other was the right lateral ventricle. Preoperative magnetic resonance imaging scans showed calcification and heterogeneous contrast enhancement in the lesions. The mass was excised surgically for the progression of symptoms. Postoperative pathologies of the tumors, combined with immunohistochemistry, revealed AT/RT. AT/RTs are often misdiagnosed as other types of brain tumors due to the lack of specific radiological features and other key characteristics. To improve awareness of AT/RT on the differential diagnosis of intracranial lesions among clinicians, we present this report and briefly summarize previous cases.

## Introduction

Atypical teratoid/rhabdoid tumor (AT/RT) is a highly malignant central nervous system (CNS) neoplasm predominantly found in children under the age of 3, and is extremely rare in adults (1, 2). In the year 1987, it was described for the first time (3). AT/RT usually occurs in posterior fossa for pediatric patients, most commonly in the cerebellum. By contrast, the most common locations in adults are the cerebral hemisphere and the sellar region (2, 4, 5). The pathological features of AT/RT are peculiar, consisting primarily of rhabdoid cells and heterogeneous portions containing mesenchymal, epithelial, and neuroectodermal cells (6).

According to the 2021 WHO Classification of CNS tumors, medulloblastoma and AT/RT were classified as embryonal tumors, and all embryonal tumors (except for cribriform neuroepithelial tumor and CNS tumor with BCL6 corepressor internal tandem duplication) were classified as Grade 4 tumor (7). Using DNA methylation and gene expression profiling, the relevant reports showed that AT/RTs are comprised of three epigenetic subgroups with distinct enhancer landscapes: ATRT-TYR, ATRT-SHH, and ATRT-MYC (8). AT/RTs are often misdiagnosed as other types of brain tumors due to the lack of specific radiological features and the heterogeneous nature of the tumor cells (6). Differential diagnoses should be noted for embryonal tumor, medulloblastoma, choroids plexus carcinoma, and high-grade glioma (9). Therefore, a diagnosis of AT/RTs requires the confirmation of specific genetic aberrations such as a loss of integrase interactor 1 (INI1) tumor suppressor gene on chromosome 22 or BRG1 gene (10, 11).

In addition, the treatment criteria (including stratification criteria) for patients with AT/RT are a controversial topic (10). The prognosis is grim, even if the treatment generally consists of surgical resection in combination with chemotherapy and radiation therapy (RT) (6). In order to better diagnose and treat AT/RT, more cases should be included in the study. In this report, we describe two patients who presented with intracranial masses in the left cerebellum and right ventricle, respectively, and show the clinical, imaging, pathological, and immunohistochemical aspects of AT/RTs.

## Case Presentation

**Figure 1:**
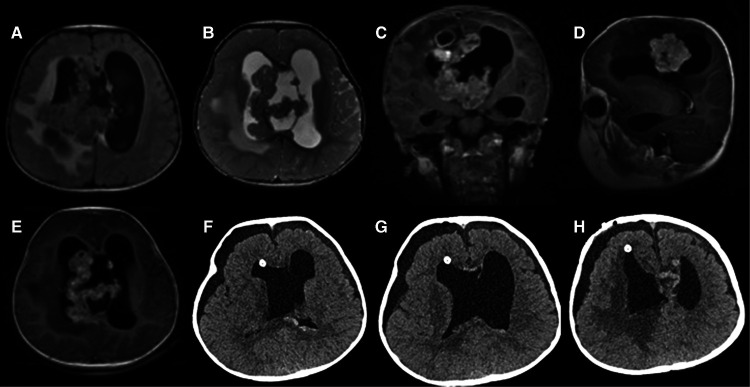
Preoperative MRI: (**A**) axial T1 image; (**B**) axial T2 image; (**C**) coronal T1-weighted image with gadolinium contrast; (**D**) sagittal T1-weighted image with gadolinium contrast; (**E**) axial T1-weighted image with gadolinium contrast; Postoperative CT: (**F–H**) axial scan.

**Figure 3:**
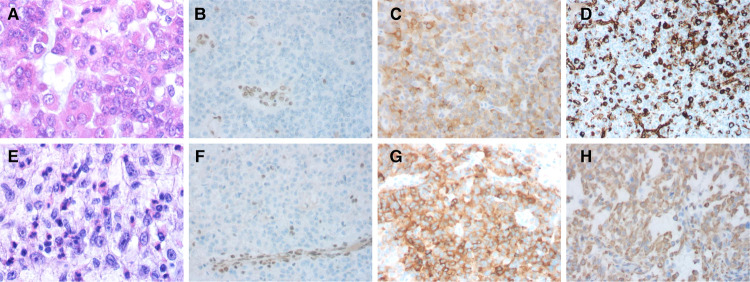
Hematoxylin and eosin stain (H&E) and immunohistochemical features of the cases. Case 1: (**A**) The majority of the tumor cells displayed eccentric round nuclei and prominent nucleolus; (**B**) IHC showing the tumor is negative for integrase interactor 1 (INI1), with vascular endothelial cell internal control being positive; (**C**) positive for Synaptophysin; and (**D**) partially positive for vimentin. Case 2: (**E**) Brisk mitotic activity is common; (**F**) Immunostaining showed a lack of INI1 protein expression in the tumor cells and apoptotic bodies; (**G**) positive for Synaptophysin; and (**H**) partially positive for vimentin.

Case 1: The patient was a 19-month-old boy with a 1-month history of claudication before admission. Neurological examination verified that the left ankle clonus and the left Babinski sign were positive. Cerebral magnetic resonance imaging (MRI) revealed a mass in the right lateral ventricle with intraventricular extension. The size of the lump was about 6 × 5 × 2 cm, which was primarily isointense on both T1- and T2-weighted MRI. Additionally, the mass appeared hyperintense on enhanced MRI, with areas of calcification and heterogeneous contrast enhancement (Figures 1A–E). The patient underwent a right frontal approach for gross total resection in supine position. To observe the yellow and soft mass, we gently slit the epidermis, subcutaneous tissue, and other tissues to expose the skull, dissociated the skull with a milling cutter, and liberated the blood vessels. Grossly, the tumor had a tender fish flesh-like appearance with necrosis. However, the tumor was tightly connected to the lateral ventricle wall and the septum pellucidum and protruded into the third ventricle through the interventricular foramen. Then, piecemeal resection was performed on the tumor. Postoperative computed tomography (CT) showed that in the bilateral lateral ventricle walls, there were several irregular nodular, strip-like high-density foci with indistinct boundaries. The tumor was completely removed (Figures 1F–H). Postoperative pathological diagnosis showed embryonic tumors with multidirectional differentiation of immature small cells, epithelial, and neuron cells and revealed abundant eosinophilic cytoplasm and eccentric nucleus with prominent nucleoli. The tumors were negative for INI1 and positive for Synaptophysin (Syn) (Figures 3A–D). The Ki-67 labeling index was 50%. A case of AT/RT was ultimately diagnosed by histopathological examination of the resected specimens. Two months after the operation, the patient received two cycles of adjuvant chemotherapy including Vincristine, Methotrexate, Leucovorin, Cytoxan, Etoposide, and Cisplatin. Unfortunately, the patient died of tumor recurrence after 15 months.

**Figure 2:**
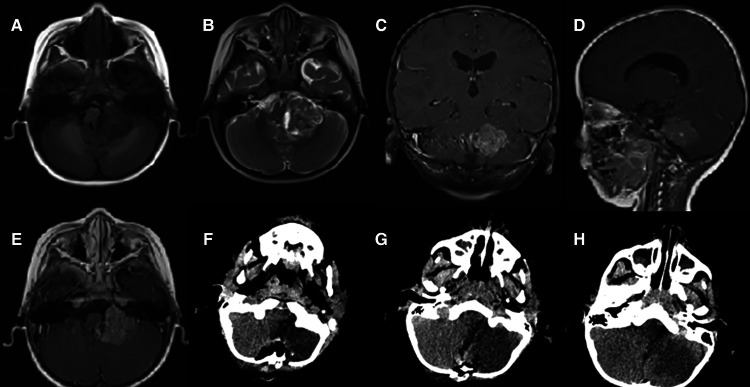
Preoperative MRI: (**A**) axial T1 image; (**B**), axial T2 image; (**C**) coronal T1-weighted image with gadolinium contrast; (**D**) sagittal T1-weighted image with gadolinium contrast; (**E**) axial T1-weighted image with gadolinium contrast; Postoperative CT: (**F**–**H**) axial scan.

Case 2: The patient was a 27-month-old boy, whose symptoms were unstable gait for a month, accompanied by nausea and vomiting for half a month. Physical examination did not reveal positive signs. An MRI revealed the presence of a mass measured 2.3 × 1.8 × 1.7 cm in the left cerebellum. MRI showed the isointense on T1-weighted images, heterogeneous iso/hyperintense on T2-weighted images and a pronounced enhancement after using an MR contrast agent (Figures 2A–E). The patient underwent a median suboccipital approach to the posterior fossa for tumor resection in the right lateral position. The epidermis and other tissues were slit progressively to reveal the tumor, just as we did in case 1. Then, we made a bone window along the squamous portion of the occipital bone. Unlike case 1, however, the tumor had a distinct capsule that separated it from the normal parenchyma. The tumor was red and soft, with abundant vascularity. A subtotal resection of the tumor was performed. Postoperative CT revealed that the left cerebellum was a low-density area, with an unclear boundary and sporadic pneumocephalus. The tumor was removed completely (Figures 2F–H). Tumor cells have eccentric nucleoli and abundant eosinophil cytoplasm. Brisk mitotic activity and apoptotic bodies are common. The tumors were partly positive for vimentin (Figures 3E–H) and the tumors were negative for INI1, Smooth Muscle Actin (SMA), and Glial Fibrillary Acidic Protein (GFAP). The Ki-67 labeling index was 40%. Postoperatively, the results of histopathological examinations demonstrated an AT/RT. After 2 months, the patient received four cycles of chemotherapy (cisplatin, cyclophosphamide, and vincristine). Despite these efforts, patient survival was less than 9 months. Notably, the two patients had no history of radiation or family history of hereditary diseases. Moreover, cerebrospinal fluid examinations for tumor markers were negative. Fortunately, no metastases occurred in the two patients. The relevant information on the patients is listed in Table 1.

**Table 1:** Clinical features, imaging findings, and therapy in two cases of AT/RTs.

	Case 1	Case 2
Age	19 m	27 m
Gender	Male	Male
Symptoms	Difficulty in walking, vomiting	Unsteady gait, nausea, vomiting
Tumor location	Right lateral ventricle	Left cerebellum
Size	6 × 5 × 3 cm	2.3 × 1.8 × 1.7 cm
CT/MRI homogeneity	Heterogeneous	Heterogeneous
CT/MRI contrast enhancement	Heterogeneous	Heterogeneous
Metastases	None	None
Radiotherapy	None	None
Chemotherapy	Yes	Yes
Follow-up	Died 15 m after operation	Died 9 m after operation

As in prior cases, the pathology of tumors showed that cells had a prominent nucleus with eccentric nucleoli and abundant cytoplasm of a somewhat “rhabdoid” morphology (6). However, pathological features cannot diagnose AT/RT. The final diagnosis of the disease still depends on the loss of nuclear INI1 expression. However, INI1 expression may be retained in a small percentage of AT/RTs, and INI1 deletion may also occur in other tumors. In our case, postoperative immunohistochemistry of all patients showed that the tumor cells were negative for INI1, OLIG2, and GFAP. The SMA was positive in the first patient. Meanwhile, immunostaining of the two patients’ tumor tissue showed that the tumor cells were positive for epithelial membrane antigen (EMA). The Ki-67 labeling indexes were 40% and 50%. The results of immunohistochemical staining are listed in Table 2.

**Table 2:** Immunohistochemical staining in two cases of AT/RTs.

Case	INI1	EMA	SMA	GFAP	CK	S-100	Syn	NSE	Neu-N	OLIG2	P53	Ki-67LI
1	−	+	+	−	+	+	+	NA	−	−	+	50%
2	−	+	−	−	+	+	+	+	−	−	+	40%

*INI1, integrase interactor 1; EMA, epithelial membrane antigen; SMA, smooth muscle actin; GFAP, glial fibrillary acidic protein; CK, cytokeratin; Syn, synaptophysin; NSE, neuron-specific enolase; NA, not available.*

## Discussion

### Predisposing

AT/RT is an extremely rare and highly aggressive CNS tumor, especially in children under 3 years of age (median age 2 years) (1, 2, 12). The prevalence of AT/RT is estimated to be approximately 1%–2% among all pediatric CNS tumors (13). The incidence of AT/RT is not high, but large sample studies are currently lacking. The prognosis of this tumor is extremely poor, with the historic median overall survival (OS) ranging from 6 to 18 months (14). The different clinical manifestations, such as vomiting, lethargy, or cranial nerve palsies, relies predominantly on the location of the tumor (15). The distribution of AT/RT is as follows: 52% in the posterior fossa (the cerebellum being the predominant site); 39% supratentorial; 5% in the pineal region; 2% in the spine; and 2% are multifocal (16). Males have a higher incidence rate than females, with the reported ratio being 3:2 to 2:1 (12). The incidence is increasing as a result of improved diagnostic techniques, especially the introduction of the biological marker INI1 (17).

### Histology

Histopathologically, AT/RT is characterized by the presence of rhabdoid cells, with or without fields resembling a typical embryonal tumor, epithelial tissue, and neoplastic mesenchyme (16–18). The rhabdoid cell has an eccentric round nucleus in an abundant eosinophilic cytoplasm and a prominent nucleolus (19). Necrosis and brisk mitotic activity are common in the rhabdoid cell (3, 19). In general, immunohistochemistry contains staining of vimentin, EMA, and other markers such as INI1, MyoD1, S-100, SMA, and GFAP (6).

### Genetics

The cellular origin of AT/RT is unknown, but inactivating mutations of the hSNF5/INI1 gene in chromosomal region 22q11.2 are regarded as a critical step in its molecular pathogenesis (20). AT/RT is characterized by a loss of the long arm of the chromosome 22, which results in a loss of the hSNF5/INI1 gene, causing the INI1 protein expression to be negative (21). The hSNF5/INI1 gene is considered as a tumor suppressor gene in peripheral AT/RTs of the CNS (22, 23). Previous studies have suggested that INI1 suppresses tumor formation by regulating cell proliferation via the Rb cell cycle checkpoint (9). But the loss of INI1 expression is not a certainty. Others have previously reported that an AT/RT retained INI1 expression staining was detected by immunohistochemistry and genetic testing. This reminds us that we cannot diagnose AT/RT merely on the basis of a loss of nuclear INI1 expression. Furthermore, they estimated that tumors that retain INI1 staining account for roughly 2% of AT/RTs. Even though the loss of nuclear INI1 expression is the most frequent surrogate marker for AT/RT diagnosis, a minor subset may have retained INI1 expression and should be evaluated for the loss of nuclear BGR1 (SMARCA4) expression. Conversely, we also bear in mind that INI1 loss may occur in other neoplasms, including poorly differentiated chordomas, and schwannomatosis-associated schwannomas (in a mosaic pattern). Therefore, correlations with morphological and clinicopathological features should be noted as well (24). In addition, the essential diagnostic criteria for AT/RT include: (1) a CNS embryonal tumor with a polyimmunophenotype AND (2) Loss of nuclear SMARCB1 (INI1) or SMARCA4 (BRG1) expression in tumor cells OR A DNA methylation profile aligned with AT/RT.

### Subgroups

Using DNA methylation and gene expression profiling, AT/RTs are comprised of three subgroups ATRT-TYR, ATRT-SHH, and ATRT-MYC (8). Furthermore, the ATRT-SHH subgroup exhibited further heterogeneity, segregating further into two subtypes: ATRT-SHH-1 and ATRT-SHH-2 (25). ATRT-TYR is characterized by tyrosinase overexpression (25, 26). The whole or partial deletion of one copy of chromosome 22, accompanied by an inactivating mutation in INI1 on the other allele, is the prototypic type of biallelic SMARCB1 inactivation in the ATRT-TYR group. ATRT-TYR patients are the youngest patient group clinically, with a median age of 12 months at diagnosis (25). Protein expression of achaete-scute homolog 1 (ASCL1) has been suggested as an immunohistochemical marker for the ATRT-SHH subgroup. But, ASCL1 may not be expressed in all ATRT-SHH patients. ASCL1-positive ATRT-SHH patients had greater OS than ASCL1-negative ATRTs (27). The ATRT-SHH subgroup was reported to be less likely to display no enhancement (28). In comparison with ATRT-TYR and ATRT-MYC, homo- or heterozygous SMARCB1 deletions are less frequently found in ATRT-SHH. ATRT-SHH is a more intermediate grouping in terms of age (median age 20 months) (25). Withholding radiotherapy in ATRT-SHH tumors appears to have no detrimental influence on OS (27). Another report found a robust trend toward longer event-free survival (EFS) and OS in ATRT-SHH tumors, with a 6-month EFS of 100% (29). MYC oncogene expression is elevated in ATRT-MYC. Point mutations are uncommon in ATRT-MYC tumors in contrast to ATRT-TYR or ATRT-SHH tumors (25). MRI studies have demonstrated that ATRT-MYC tumors are characterized by the presence of intense peritumoral edema (28). The median age of ATRT-MYC patients is significantly higher than that of the other subgroups (27 months) (25). Both ATRT-TYR and ATRT-SHH tumors tend to have focal aberrations in SMARCB1, whereas ATRT-MYC tumors tend to have broad deletions affecting Chr22q11.2. Unlike ATRT-SHH cancers, ATRT-MYC and ATRT-TYR tumors have a strong reliance on receptor tyrosine kinase pathways (27).

### Management

At present, it is indistinct to formulate a vintage treatment for AT/RT. Therapeutic strategies of AT/RT are largely dependent on the age of the patient, the location of the tumor in the CNS, and the stage of the disease at the time of diagnosis (25). In general, surgical resection of AT/RT is the principal treatment. Surgical treatment generally guarantees most of the removal of the tumor according to the relationship between the location of the tumor and the blood vessels in the surrounding tissues. Taking into account the blood vessels around the tumor, especially the tumor in difficult location, angiography and preoperative embolization may yield some effects on surgery (30). Adjuvant therapy includes RT, high-dose chemotherapy, and autologous stem cell transplantation (12, 31). RT is an effective treatment but can be avoided in patients under 3 years of age due to long-term neurocognitive sequelae. Patients receiving RT earlier in the treatment plan may not experience early relapse or progression during induction chemotherapy and may show improvement in outcomes (12, 32). In addition, it has been demonstrated that antisense-mediated downregulation of insulin-like growth factor I receptor (IGF-IR) results in sensitization to doxorubicin and cisplatin (18). The use of high-dose chemotherapy and autologous stem cell transplantation (HDCT/auto-SCT) has shown clinical benefits in children with brain tumors (33). Moreover, existing literature has shown that a tumor vaccination strategy using lysate-loaded autologous dendritic cells is feasible and safe even in small children with AT/RT (34). When the prognosis is poor, people may try to regain control through the use of alternative therapies, including dietary changes. Complementary and alternative medicine (CAM) may not be limited to prayer, spiritual healing, vitamins, herbs, and dietary changes (15).

### Limitation

However, there are some limitations in this report. First, the number of cases included in the report is relatively small. So far, there is still a lack of large sample studies, and no mature therapeutic plan has been formed. Furthermore, the figures of postoperative MRI images are missing. Finally, according to multicenter data, AT/RT is still a rare tumor with poor prognosis in children. In general, most of the patients died of local relapse, metastatic lesions through the subarachnoid space, or distant metastasis (35).

## Conclusion

AT/RT is a rare disease condition, and a preoperative diagnosis is difficult without an adequate knowledge of the disease. To date, despite multiple advanced diagnoses and treatments being available, the prognosis of patients with AT/RT remains grim. More AT/RT case reports should be included, which will lead to greater diagnostic accuracy as well as improvement in treatment and prognosis. Due to its aggressiveness and poor prognosis, AT/RT cannot be ruled out in the differential diagnosis of intracranial lesions. The case reports replenish the existing literature with new knowledge and hopefully will draw the attention of doctors to the rare tumor.

## Data Availability

The original contributions presented in the study are included in the article/supplementary material; further inquiries can be directed to the corresponding author/s.
